# Multiplex genotyping system for efficient inference of matrilineal genetic ancestry with continental resolution

**DOI:** 10.1186/2041-2223-2-6

**Published:** 2011-03-23

**Authors:** Mannis van Oven, Mark Vermeulen, Manfred Kayser

**Affiliations:** 1 Department of Forensic Molecular Biology, Erasmus MC University Medical Center Rotterdam, 3000 CA Rotterdam, The Netherlands

## Abstract

**Background:**

In recent years, phylogeographic studies have produced detailed knowledge on the worldwide distribution of mitochondrial DNA (mtDNA) variants, linking specific clades of the mtDNA phylogeny with certain geographic areas. However, a multiplex genotyping system for the detection of the mtDNA haplogroups of major continental distribution that would be desirable for efficient DNA-based bio-geographic ancestry testing in various applications is still missing.

**Results:**

Three multiplex genotyping assays, based on single-base primer extension technology, were developed targeting a total of 36 coding-region mtDNA variants that together differentiate 43 matrilineal haplo-/paragroups. These include the major diagnostic haplogroups for Africa, Western Eurasia, Eastern Eurasia and Native America. The assays show high sensitivity with respect to the amount of template DNA: successful amplification could still be obtained when using as little as 4 pg of genomic DNA and the technology is suitable for medium-throughput analyses.

**Conclusions:**

We introduce an efficient and sensitive multiplex genotyping system for bio-geographic ancestry inference from mtDNA that provides resolution on the continental level. The method can be applied in forensics, to aid tracing unknown suspects, as well as in population studies, genealogy and personal ancestry testing. For more complete inferences of overall bio-geographic ancestry from DNA, the mtDNA system provided here can be combined with multiplex systems for suitable autosomal and, in the case of males, Y-chromosomal ancestry-sensitive DNA markers.

## Background

Establishing the geographic region of a person's genetic origin - also called bio-geographic ancestry - is of forensic relevance when the short tandem repeat (STR) profile of trace DNA found at a crime scene does not match that of a suspect or does not yield any matches in a criminal DNA database because it may provide investigative leads to finding unknown persons [1]. Similarly, such information can be useful for locating antemortem samples or putative relatives of unidentified body remains, including disaster victim identification [2]. Furthermore, inferring geographic information from DNA data is important in population history studies [3,4] and has gained attention in the growing field of personal ancestry testing [5,6].

Several years of intensive research into the understanding of the geographic distribution of human genetic diversity present in the non-recombining mitochondrial genome and respective parts of the Y-chromosome (NRY), mostly for population history purposes, have produced an immense body of knowledge allowing us to pick specific mtDNA and NRY markers with restricted (sub)continental distributions [4,7,8]. MtDNA is especially useful for forensic application due to its high copy number (hundreds to thousands of copies per cell) and small size (16.6 kb), which allows the analysis of small amounts of degraded DNA often encountered in crime-scene situations [9]. Although mtDNA only reveals information about matrilineal ancestry, it can be seen as a first step toward a more comprehensive picture of personal ancestry when combined with suitable NRY and autosomal DNA evidence [10,11]. Furthermore, investigating the geographic origin of mtDNA in comparison to that of the Y-chromosome in a population can also reveal insights into sex-biased aspects of human population history such as those caused by patri- or matrilocal residence patterns [12].

In human population genetics studies, the typical approach for mtDNA analysis consists of sequencing the first hypervariable segment (HVS1), sometimes in combination with HVS2, within the non-coding control region (see, for example [13,14]), whereas in forensics it has nowadays become standard practice to sequence the entire control region [15]. Although haplogroup inference from HVS sequence data is possible for many mtDNA haplogroups, not all haplogroups present suitable diagnostic variants in HVS1 and/or HVS2 that allow an unequivocal assignment. In such cases, simple nucleotide polymorphisms (SNPs; i.e. single-nucleotide polymorphisms as well as small insertions and deletions) from the coding region of mtDNA are required in order to establish the haplogroup status. Moreover, because SNP typing assays are usually more sensitive and consume less DNA than sequencing, in many cases it might be desirable to perform SNP genotyping alone (in the absence of HVS data) or prior to HVS sequencing [16,17].

Several mtDNA SNP multiplex assays have already been developed focussing on particular geographic subregions (see, for example, [18]) or on the dissection of particular haplogroups (see, for example, [19]). However, what is missing so far is an mtDNA SNP multiplex system that includes the mtDNA haplogroups of major continental distribution. We describe a sensitive genotyping system based on single-base primer extension technology, consisting of three independent multiplex assays that together include 36 SNPs determining 43 mtDNA haplo-/paragroups that allow the inference of matrilineal bio-geographic ancestry at the level of continental resolution.

## Results and discussion

### Multiplexes and targeted haplogroups

**Figure 1:**
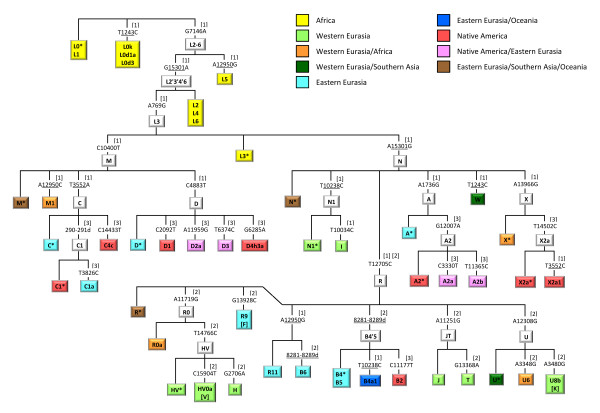
**Overall phylogenetic scheme of targeted mtSNPs with geographic haplogroup classification**. The combined use of the three multiplex assays allows any person's mtDNA to be classified into one of the colour-labelled haplogroups. Colours correspond to the geographic origin of the haplogroups as indicated. SNP position numbers are relative to the revised Cambridge Reference Sequence (rCRS). Deletion mutations are denoted by the suffix 'd'. Recurrent SNPs are underlined. The numbers 1, 2 or 3 in square brackets shown for each SNP refer to the respective multiplex assay in which the SNP is included. Note: haplogroups F, K and V are encompassed within R9, U8b and HV0a, respectively, as indicated because this does not follow logically from the nomenclature.

**Figure 2:**
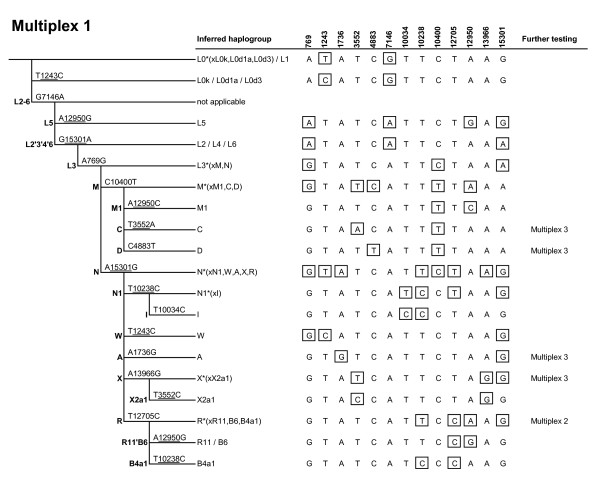
**Marker phylogeny and haplogroup-defining genotypes of Multiplex 1**. Recurrent SNPs are underlined. Boxed alleles indicate for each haplogroup those SNPs that are minimally required to define that haplogroup. If additional genotyping is required for more detailed haplogroup inference, the respective additional multiplex to be genotyped subsequently is noted.

**Figure 3:**
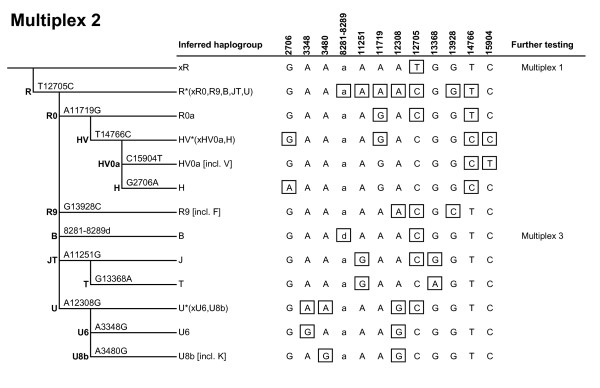
**Marker phylogeny and haplogroup-defining genotypes of Multiplex 2**. Boxed alleles indicate for each haplogroup those SNPs that are minimally required to define that haplogroup. The allelic states of deletion polymorphism 8281-8289 are denoted as 'a' (ancestral) and 'd' (deletion), respectively. If additional genotyping is required for more detailed haplogroup inference, the respective additional multiplex to be genotyped subsequently is noted.

**Figure 4:**
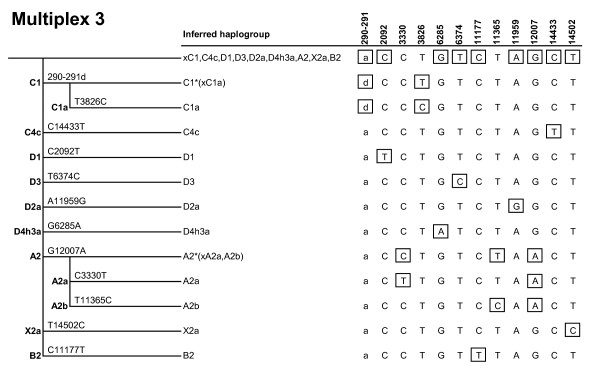
**Marker phylogeny and haplogroup-defining genotypes of Multiplex 3**. Boxed alleles indicate for each haplogroup those SNPs that are minimally required to define that haplogroup. The allelic states of deletion polymorphism 290-291 are denoted as 'a' (ancestral) and 'd' (deletion), respectively.

MtDNA coding-region SNPs defining the major haplogroups that occur in Africa, Western Eurasia, Eastern Eurasia and Native America were carefully selected (Figure 1) and combined into three multiplex genotyping assays (Figures 2, 3, 4) each consisting of a polymerase chain reaction (PCR) amplification step and a subsequent single-base primer extension step (Tables 1, 2, 3). The haplogroups detectable with Multiplex 1 and 2 are broadly similar to those typed by the Genographic Project [20] with some noticeable exceptions. Multiplex 1 (Figure 2) was designed to target haplogroups L0/L1, L2/L4/L6, L3, M, M1, C, D, N, N1, I, W, A, X and R. Due to the homoplasy of some of the selected markers in the worldwide mtDNA phylogeny [7], Multiplex 1 can additionally detect some (relatively rare) haplogroups that were not originally intended, namely L0k/L0d1a/L0d3, L5, X2a1, R11/B6 and B4a1. The hierarchical organization of the mitochondrial SNPs in Multiplex 1 ensures that all these haplogroups, intended and unintended, are well differentiable (Figure 2). Some haplogroups are only identified with Multiplex 1 on a broad level and, in those cases, additional genotyping with Multiplex 2 or 3 is needed to achieve further haplogroup resolution and final geographic inferences. Multiplex 2 (Figure 3) targets haplogroup R and haplogroups nested within R, namely R0, HV, HV0a (which includes V), H, R9 (which includes F), B, J, T, U, U6 and U8b (which includes K). A notable difference with the Genographic Project SNP panel [20] is that we included in our multiplexes haplogroups M1 and U6 which have a predominantly African distribution, probably due to back-migration events to Africa [21]. As such, Multiplex 1 and 2 together offer a convenient method for the classification of unknown mtDNAs into any of the major worldwide mtDNA haplogroups. However, they do not allow for the differentiation of the Native American subsets of otherwise Eastern Eurasian haplogroups A, B, C and D and Western Eurasian/African haplogroup X. Therefore, we designed a third assay, Multiplex 3 (Figure 4), which specifically aims at detecting the Native American haplogroups A2, B2, C1, C4c, D1, D4h3a and X2a, as well as Eskimo/Siberian haplogroups A2a, A2b, D2a and D3 and Eastern Eurasian haplogroup C1a [22]. Together, the three multiplexes include 36 different coding-region mtDNA SNPs (of which 34 are single-nucleotide transitions/transversions and two are small insertion/deletion polymorphisms). It should be noted that, despite the fact that haplogroups M1, C and D within macrohaplogroup M, haplogroups N1, A, W and X within macrohaplogroup N, and haplogroups R0, R9, B, JT and U within macrohaplogroup R, can be detected with the method, much of the Southern Asian, East/Southeast Asian and Oceanic variation within M, N and R remains unresolved (denoted as M*, N* and R*, respectively, in Figure 1). However, this is inevitable given the large number of independent haplogroups descending from M, N and R but it can be overcome by developing additional multiplex assays that specifically target the relevant subhaplogroups for those regions.

**Table 1:** Primer details for Multiplex 1.

	PCR amplification				Single-base extension				
	
Site	Primer sequences (5'-3')		Concentration (μM)	Amplicon size (bp)	Primer sequence (5'-3') (5' aspecific tail in lowercase italics)	Concentration (μM)	Length (nt)	Orientation	Alleles (dye)
769	F	ACATCACCCCATAAACAAATAGG	1.000	158	*act(gact)*_*10 *_CGTTTTGAGCTGCATTG	2.000	60	R	A (red), G (yellow)
	R	AGCGTTTTGAGCTGCATTG	1.000						

1243	F	AATCGATAAACCCCGATCAA	0.040	93	*actgact *CGATCAACCTCACCACC	0.150	24	F	C (yellow), T (red)
	R	TGCGCTTACTTTGTAGCCTTC	0.040						

1736	F	GCTAAACCTAGCCCCAAACC	0.200	106	*t(gact)*_*2*_*gac *TCAATTTCTATCGCCTATACTTTAT	0.150	37	R	A (red), G (yellow)
	R	CTATTGCGCCAGGTTTCAAT	0.200						

3552	F	CGCTGACGCCATAAAACTCT	4.000	128	*actgact *AGGGGGGTTCATAGTAGAAG	4.000	27	R	A (red), C (blue), T (green)
	R	GTATGGGGAGGGGGGTTC	4.000						

4883	F	TGACATCCGGCCTGCTT	0.075	114	*(gact)*_*4*_*g *CATGACAAAAACTAGCCCC	0.300	36	F	C (yellow), T (red)
	R	TGGATAAGATTGAGAGAGTGAGGA	0.075						

7146	F	AGACCAAACCTACGCCAAAA	0.150	130	*ct(gact)*_*9*_*ga *TACGCCAAAATCCATTTC	0.300	58	F	A (green), G (blue)
	R	GGTGTATGCATCGGGGTAGT	0.150						

10034	F	TCTCCATCTATTGATGAGGGTCT	0.300	108	*ct(gact)*_*4*_*g *GTACCGTTAACTTCCAATTAACTAG	0.150	44	F	C (yellow), T (red)
	R	TTAAGGCGAAGTTTATTACTCTTTTT	0.300						

10238	F	GCGTCCCTTTCTCCATAAAA	0.250	80	*ct(gact)*_*4*_*gac *CTCCATAAAATTCTTCTTAGTAGCTAT	0.300	48	F	C (yellow), T (red)
	R	GGGTAAAAGGAGGGCAATTT	0.250						

10400	F	GCCCTAAGTCTGGCCTATGA	0.150	90	*ct(gact)*_*5*_*gac *CGTTTTGTTTAAACTATATACCAATTC	0.300	52	R	C (blue), T (green)
	R	TGAGTCGAAATCATTCGTTTT	0.150						

12705	F	CCCAAACATTAATCAGTTCTTCAA	0.180	102	*t(gact)*_*6*_*g *TTAATCAGTTCTTCAAATATCTACTCAT	0.150	54	F	C (yellow), T (red)
	R	TCTCAGCCGATGAACAGTTG	0.180						

12950	F	TCCTCGCCTTAGCATGATTT	0.200	101	*act(gact)*_*11 *_TGAGGCTTGGATTAGCG	0.500	64	R	A (red), C (blue), G (yellow)
	R	GAGGCCTAGTAGTGGGGTGA	0.200						

13966	F	ACCGCACAATCCCCTATCTA	0.250	132	*ct(gact)*_*13 *_GCAGGTTTTGGCTCG	0.500	69	R	A (red), G (yellow)
	R	AGGTGATGATGGAGGTGGAG	0.250						

15301	F	CCACCCTCACACGATTCTTT	0.080	119	*ct(gact)*_*4 *_ATTCTTTACCTTTCACTTCATCTT	0.150	42	F	A (green), G (blue)
	R	GGTGATTCCTAGGGGGTTGT	0.080						

**Table 2:** Primer details for Multiplex 2.

	PCR amplification				Single-base extension				
	
Site	Primer sequences (5'-3')		Concentration (μM)	Amplicon size (bp)	Primer sequence (5'-3') (5' aspecific tail in lowercase italics)	Concentration (μM)	Length (nt)	Orientation	Alleles (dye)
2706	F	CGAGGGTTCAGCTGTCTCTT	0.040	88	*(gact)*_*10 *_GTCTTCTCGTCTTGCTGTGT	0.100	60	R	A (red), G (yellow)
	R	AGGGTCTTCTCGTCTTGCTG	0.040						

3348	F	CAGTCAGAGGTTCAATTCCTCTT	0.200	142	*act(gact)*_*11*_*g *GGAATGCCATTGCGAT	0.200	64	R	A (red), G (yellow)
	R	GGGCCTTTGCGTAGTTGTAT	0.200						

3480	F	CGCTGACGCCATAAAACTCT	0.120	120	*act(gact)*_*2*_*ga *GCCATAAAACTCTTCACCAA	0.150	33	F	A (green), G (blue)
	R	AGGGGGGTTCATAGTAGAAG	0.120						

8281-8289	F	GAAATCTGTGGAGCAAACCAC	0.500	179/170	*(gact)*_*2*_*g *CCCTATAGCACCCCCTCTA	0.700	28	F	a (yellow), d (blue)*
	R	AGAGGTGTTGGTTCTCTTAATCTTT	0.500						

11251	F	TGAACGCAGGCACATACTTC	0.100	92	*t(gact)*_*5*_*gac *CCCCTACTCATCGCACT	0.050	41	F	A (green), G (blue)
	R	TGAGCCTAGGGTGTTGTGAG	0.100						

11719	F	GGCGCAGTCATTCTCATAATC	0.100	85	*(gact)*_*6 *_GCAGAATAGTAATGAGGATGTAAG	0.150	48	R	A (red), G (yellow)
	R	TGTGAGTGCGTTCGTAGTTTG	0.100						

12308	F	CAGCTATCCATTGGTCTTAGGC	2.000	169	*t(gact)*_*9*_*gac *TGGTCTTAGGCCCCAA	3.000	56	F	A (green), G (blue)
	R	GATTTTACATAATGGGGGTATGAGT	2.000						

12705	F	ACTTCTCCATAATATTCATCCCTGT	1.300	184	*act(gact)*_*5*_*g *TTAATCAGTTCTTCAAATATCTACTCAT	0.800	52	F	C (yellow), T (red)
	R	TCTCAGCCGATGAACAGTTG	1.300						

13368	F	CGCCTTCTTCAAAGCCATAC	0.250	127	*ct(gact)*_*2*_*gac *TAAGGTTGTGGATGATGGA	0.300	32	R	A (red), G (yellow)
	R	GGTGAGGGAGGTTGAAGTGA	0.250						

13928	F	CAGCCCTAGACCTCAACTACCT	0.040	119	*ct(gact)*_*5*_*ga *AACATACTCGGATTCTACCCTA	0.040	46	F	C (yellow), G (blue)
	R	ATAGGGGATTGTGCGGTGT	0.040						

14766	F	TCAACTACAAGAACACCAATGACC	0.050	109	*c *GACCCCAATACGCAAAA	0.150	18	F	C (yellow), T (red)
	R	ATCATGCGGAGATGTTGGAT	0.050						

15904	F	CATCCGTACTATACTTCACAACAATC	1.000	184	*act(gact)*_*4 *_GGCCTGTCCTTGTAGTATAAA	0.600	40	F	C (yellow), T (red)
	R	GGTGCTAATGGTGGAGTTAAAGA	1.000						

* a, ancestral; d, deletion.

**Table 3:** Primer details for Multiplex 3.

	PCR amplification				Single-base extension				
	
Site	Primer sequences (5'-3')		Concentration (μM)	Amplicon size (bp)	Primer sequence (5'-3') (5' aspecific tail in lowercase italics)	Concentration (μM)	Length (nt)	Orientation	Alleles (dye)
290-291	F	CGCTTTCCACACAGACATCA	0.350	94/92	*(gact)*_*6*_*gac *CCACACAGACATCATAACAAAA	0.350	49	F	a (green), d (red)*
	R	GGGTTTGGCAGAGATGTGTT	0.350						

2092	F	TTGCCCACAGAACCCTCTAA	0.300	141	*act(gact)*_*3*_*ga *CTCTAAATCCCCTTGTAAATTTAA	0.400	41	F	C (yellow), T (red)
	R	AATTGGTGGCTGCTTTTAGG	0.300						

3330	F	CAGTCAGAGGTTCAATTCCTCTT	0.100	142	*act(gact)*_*7 *_CGATTAGAATGGGTACAATGAG	0.150	53	R	C (blue), T (green)
	R	GGGCCTTTGCGTAGTTGTAT	0.100						

3826	F	TGAAGTCACCCTAGCCATCA	0.200	168	*ct(gact)*_*3 *_GGTCATGATGGCAGGAGTA	0.200	33	R	C (blue), T (green)
	R	AAGGGGGTTCGGTTGGT	0.200						

6285	F	GAGCAGGAACAGGTTGAACA	0.300	237	*actgactga *CCTGCTAAGGGAGGGTAGA	0.450	28	R	A (red), G (yellow)
	R	GGAGAGATAGGAGAAGTAGGACTGC	0.300						

6374		same amplicon as 6285			*ctgact *TTGATGGCCCCTAAGAT	0.300	23	R	C (blue), T (green)

11177	F	TTCACAGCCACAGAACTAATCA	0.200	164	*ct(gact)*_*10*_*gac *GCGTTCAGGCGTTCTG	0.600	61	R	C (blue), T (green)
	R	AGTGCGATGAGTAGGGGAAG	0.200						

11365	F	CACCCTAGGCTCACTAAACATTC	0.100	160	*t(gact)*_*9*_*g *CTTAATATGACTAGCTTACACAATAGC	0.100	65	F	C (yellow), T (red)
	R	TTCGACATGGGCTTTAGGG	0.100						

11959	F	CCCCCACTATTAACCTACTGG	0.200	190	*act(gact)*_*10*_*gac *CTCCTACTTACAGGACTCAACAT	0.150	69	F	A (green), G (blue)
	R	TCTCGTGTGAATGAGGGTTTT	0.200						

12007		same amplicon as 11959			*ct(gact)*_*9*_*gac *GGTGGGTGAGTGAGCC	0.300	57	R	A (red), G (yellow)

14433	F	CTCCATCGCTAACCCCACTA	0.400	170	*(gact)*_*6 *_GCTATTGAGGAGTATCCTGAG	0.300	45	R	C (blue), T (green)
	R	TTCTGAATTTTGGGGGAGGT	0.400						

14502		same amplicon as 14433			*act(gact)*_*3*_*ga *CATCATTCCCCCTAAATAAA	0.450	37	F	C (yellow), T (red)

* a, ancestral; d, deletion.

### Design and optimization

**Figure 5:**
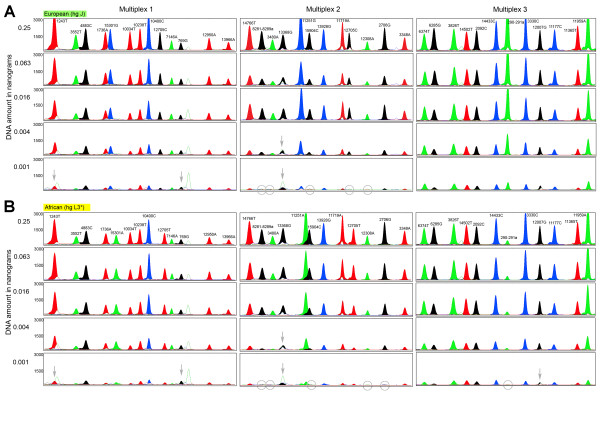
**Electropherograms of Multiplex 1-3 for a European and an African individual, using varying amounts of initial DNA template**. (A) European individual of haplogroup J; (B) African individual of haplogroup L3*(xM,N). The three multiplex assays were each performed on five different starting amounts of DNA template, ranging from 0.25 ng to 0.001 ng. Grey circles indicate marker dropouts that occur at the very low DNA concentration whereas grey arrows indicate cases where allele calling becomes difficult due to artefacts that come up at the low DNA concentrations.

**Figure 6:**
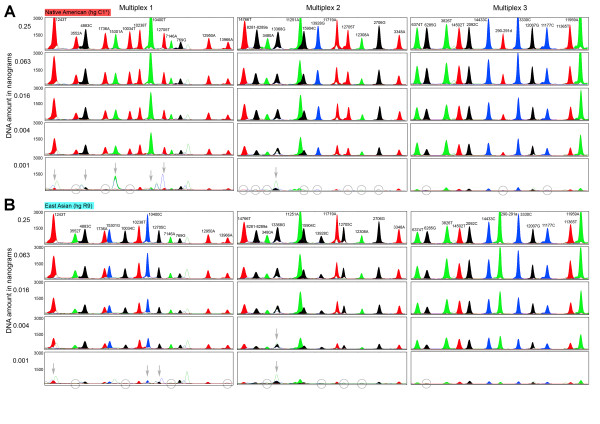
**Electropherograms of Multiplex 1-3 for a Native American and an East Asian individual, using varying amounts of initial DNA template**. (A) Native American individual of haplogroup C1*(xC1a); (B) East Asian individual of haplogroup R9. The three multiplex assays were each performed on five different starting amounts of DNA template, ranging from 0.25 ng to 0.001 ng. Grey circles indicate marker dropouts that occur at the very low DNA concentration whereas grey arrows indicate cases where allele calling becomes difficult due to artefacts that come up at the low DNA concentrations.

The successful dessign of a useful multiplex single-base extension assay requires careful consideration of the SNPs and their PCR amplification primers as well as extension primers, followed by extensive laboratory testing [23]. One criterion of SNP selection was the overall level of homoplasy of the marker in the entire mtDNA phylogeny [7]. For each haplogroup, one or several defining SNPs are available; in the latter case care was taken to select the more stable (phylogenetically less recurrent) SNP sites. Nevertheless, some of the selected SNPs do occur more than once in the phylogeny (underlined in Figure 1) as discussed above. Notably, Multiplex 1 contains two tri-allelic SNPs: nucleotide position (np) 3552 is either a T (ancestral state), an A (haplogroup C), or a C (haplogroup X2a1); and np 12950 is either an A (ancestral state), a C (haplogroup M1) or a G (haplogroups L5, R11 and B6). Primer design using Primer3Plus [24] considered small amplicon size and avoided numt amplification [25]. The compatibility of primers within the same multiplex was checked with AutoDimer [26], especially avoiding 3' end complementarities. Amplicon sizes were kept small, ranging from 80 to 237 bp with an average of 133 bp (Tables 1, 2, 3), in order to facilitate the amplification of (partially) degraded DNA typically encountered in forensic settings as well as in population history studies when using difficult source materials (for example, ancient DNA). All primers were first tested in singleplex before combining them in a multiplex. Primers that showed substantial artifacts were replaced by alternatively designed primers. In order to ensure electrophoretic separation of extension primer products, extension primers within the same multiplex were given different lengths by adding 5' non-homologous (poly)GACT tails (Tables 1, 2, 3). Peak heights in the electropherograms (Figures 5, 6) were balanced by adjusting primer concentrations in the PCR and extension reactions (Tables 1, 2, 3).

### Haplogroup distribution and inferring bio-geographic ancestry

The labels used to describe the geographic affiliations of the haplogroups (Figure 1) mostly correspond to one of four regions or continents of the world, namely Africa, Western Eurasia, Eastern Eurasia and Native America, consistent with the terminology used in human genetics and anthropology literature. With some haplogroups, however, only combined regions can be inferred, namely Western Eurasia/Africa, Western Eurasia/Southern Asia, Eastern Eurasia/Oceania, Native America/Eastern Eurasia and Eastern Eurasia/Southern Asia/Oceania (Figure 1). While these geographic designations are convenient descriptors of the 'center of gravity' of haplogroup occurrence, it is important to keep in mind that, instead of sharp genetic borders, there exist transition areas between continents. Populations from the Middle East, for example, carry a considerable portion of African mtDNA lineages [27]. Similarly, Northern Africa has a relatively large portion of Western Eurasian mtDNA lineages [28,29]. In addition, the Central Asian mtDNA pool is composed of Western Eurasian, Eastern Eurasian and, to a lesser extent, also Southern Asian components [30,31]

Furthermore, one should be aware that traditional distribution patterns of genetic variation, including mtDNA, may have been affected by (evolutionary recent) migration/admixture events, including as a result of colonialism, so that some populations carry portions of ancestry from multiple geographic regions. The most prominent case is, perhaps, the American continent where, due to colonization by Europeans which started around the beginning of the 16th century and the subsequent European introduction of African slaves, the current population carries a mixture of Native American, Western Eurasian and African mtDNA lineages, in varying proportions depending on the subpopulation [10,11,14,32,33]. Other well-known cases include Madagascar (African and Eastern Eurasian lineages) [13], and coastal/island parts of Near Oceania, as well as all of Remote Oceania (Oceanic and Eastern Eurasian lineages) [34]. In addition, groups of more or less recent immigrants often carry a mixture of 'native' lineages and lineages typical from the area to which they moved. For example, Polish Roma, having an ultimate origin in India, harbour both Southern Asian and Western Eurasian mtDNA variants [35]. Finally, rare cases have been reported where European individuals carried African mtDNA haplogroups without being aware of any African ancestry [36]. Therefore, for any bio-geographic ancestry prediction purposes, mtDNA evidence should be interpreted in the context of the relevant local demographic history. Also, because mtDNA only reflects the matrilineal portion of a person's genetic ancestry, ideally the markers should be combined with evidence obtained from autosomal and/or (when dealing with male DNA) Y-chromosome markers, to obtain a more accurate picture of a person's overall ancestry.

### Sensitivity testing

In order to establish the sensitivity of our multiplex assays we performed tests with different starting amounts of genomic DNA, ranging from 25 ng to 1 pg of template DNA, for four individuals originating from different continents and with respective diagnostic haplogroups: a European with haplogroup J; an African with L3*(xM,N); a Native American with C1*(xC1a); and an East Asian with R9 (Figures 5, 6). This enabled us to monitor the behaviour of the different SNP alleles with decreasing amounts of template DNA. Overall, we observed high sensitivity and basically full profiles could be obtained with all three multiplexes for all four individuals with as little as 4 pg of DNA template (with the only exception of 13368 in Multiplex 2 that sometimes caused difficulties in allele calling with 4 pg and lower). Marker dropouts for some SNPs in all the individuals and all three multiplexes (except for Multiplex 1 in the European and the African sample and with Multiplex 3 in the European) started to occur only at the 1 pg level, as well as allele-calling difficulties for some other SNPs in all three multiplexes (Figures 5, 6). The achieved sensitivity is similar to that of two previously published mtDNA multiplex assays [18,37] but, presumably, higher than that of many other published mtDNA multiplexes which typically require 1-10 ng DNA (for example [19,38-40]; although many such studies do not provide details on sensitivity). Furthermore, the achieved sensitivity of our assays is significantly higher than that of commercially available STR multiplexes [41-43], which can be expected due to the higher relative abundance of mtDNA as compared to nuclear DNA. When working with ancient DNA or forensic trace DNA, it might be useful to quantify the amount of human DNA prior to genotyping because, in such situations, human DNA often represents only a fraction of total DNA due to the presence of non-human (for example, bacterial, fungal, or others) DNA.

### Illustration of the method application

In order to illustrate the reliability of our method in inferring bio-geographic ancestry from mtDNA, we compared in worldwide individuals, their haplogroup status as determined from full mtDNA sequence data and their population affiliation known from the sampling region, with the haplogroup and corresponding geographic information obtainable from our multiplex SNP assays (Table 4). The data used for this purpose consisted of 75 samples from the Centre d'Etude du Polymorphisme Humain-Human Genome Diversity Project (CEPH-HGDP) panel [44] for which entire mitochondrial genome sequences are available [45]. From the full mtDNA sequences we extracted the alleles of those SNP sites that are included in our assays and used the resulting genotypes to infer haplogroups and respective geographic regions of matrilineal origin. In all cases, the haplogroups inferable by our assays were consistent with the full sequence-based haplogroups (although a more detailed haplogroup assignment could be achieved from the sequence data as expected); accordingly, the regions of bio-geographic ancestry derived from the assay-inferable haplogroups were in agreement with the individuals' sampling origins (Table 4). For example, sample HGDP01076 is an individual from Sardinia (Italy) whose full mtDNA sequence can be classified as haplogroup J2b1a; our assays would predict the haplogroup of this person as J with Western Eurasian geographic origin. Notably, the HGDP samples from Pakistan exhibit both Western Eurasian and Southern Asian haplogroups (for example, HGDP00163 belongs to Western Eurasian haplogroup H2a and HGDP00165 belongs to Southern Asian haplogroup M30), consistent with previous observations (see Discussion above). Similarly, the Bedouin samples belong to both African as well as Western Eurasian haplogroups.

**Table 4:** Established haplogroup and geographic origin versus haplogroup and geographic origin as inferable by Multiplex 1-3, for 75 CEPH-HGDP individuals.

GenBank accession	HGDP ID	HGDP population/sampling region	Full sequence-based haplogroup*	Multiplex-inferred haplogroup	Multiplex-inferred matrilineal origin
EU597551.1	HGDP00003	Brahui (Pakistan)	R2	R*(xR0,R9,R11,B,JT,U)	E Eurasia/S Asia/Oceania
EU597563.1	HGDP00005	Brahui (Pakistan)	M5a2a	M*(xM1,C,D)	E Eurasia/S Asia/Oceania
EU597574.1	HGDP00007	Brahui (Pakistan)	H2b	H	W Eurasia
EU597492.1	HGDP00163	Sindhi (Pakistan)	H2a	H	W Eurasia
EU597504.1	HGDP00165	Sindhi (Pakistan)	M30	M*(xM1,C,D)	E Eurasia/S Asia/Oceania
EU597516.1	HGDP00167	Sindhi (Pakistan)	M2a1a	M*(xM1,C,D)	E Eurasia/S Asia/Oceania
EU597528.1	HGDP00213	Pathan (Pakistan)	K2a5	U8b [incl. K]	W Eurasia
EU597540.1	HGDP00214	Pathan (Pakistan)	U9b1	U*(xU6,U8b)	W Eurasia/S Asia
EU597552.1	HGDP00216	Pathan (Pakistan)	J1d	J	W Eurasia
EU597564.1	HGDP00277	Kalash (Pakistan)	U4a1	U*(xU6,U8b)	W Eurasia/S Asia
EU597575.1	HGDP00279	Kalash (Pakistan)	U4a1	U*(xU6,U8b)	W Eurasia/S Asia
EU597493.1	HGDP00281	Kalash (Pakistan)	R0a	R0a	W Eurasia/Africa
EU597489.1	HGDP00458	Biaka_Pygmies (CAR)	L1c1a2a1	L0*(xL0k,L0d1a,L0d3)/L1	Africa
EU597501.1	HGDP00459	Biaka_Pygmies (CAR)	L1c1a1a1b1	L0*(xL0k,L0d1a,L0d3)/L1	Africa
EU597513.1	HGDP00460	Biaka_Pygmies (CAR)	L1c1a1a1b	L0*(xL0k,L0d1a,L0d3)/L1	Africa
EU597525.1	HGDP00463	Mbuti_Pygmies (DRC)	L2a2b1	L2/L4/L6	Africa
EU597537.1	HGDP00467	Mbuti_Pygmies (DRC)	L0a2b	L0*(xL0k,L0d1a,L0d3)/L1	Africa
EU597549.1	HGDP00468	Mbuti_Pygmies (DRC)	L2a2b1	L2/L4/L6	Africa
EU597495.1	HGDP00545	Papuan (New Guinea)	Q1	M*(xM1,C,D)	E Eurasia/S Asia/Oceania
EU597507.1	HGDP00546	Papuan (New Guinea)	P1d1	R*(xR0,R9,R11,B,JT,U)	E Eurasia/S Asia/Oceania
EU597519.1	HGDP00547	Papuan (New Guinea)	Q3a1	M*(xM1,C,D)	E Eurasia/S Asia/Oceania
EU597573.1	HGDP00608	Bedouin (Israel)	I5a1	I	W Eurasia
EU597491.1	HGDP00609	Bedouin (Israel)	L2a1	L2/L4/L6	Africa
EU597503.1	HGDP00610	Bedouin (Israel)	U7	U*(xU6,U8b)	W Eurasia/S Asia
EU597515.1	HGDP00675	Palestinian	H13a1	H	W Eurasia
EU597527.1	HGDP00676	Palestinian	U5a1a1	U*(xU6,U8b)	W Eurasia/S Asia
EU597539.1	HGDP00677	Palestinian	HV0c	HV*(xHV0a,H)	W Eurasia
EU597569.1	HGDP00709	Colombian	B2e	B2	Native America
EU597580.1	HGDP00710	Colombian	B2	B2	Native America
EU597554.1	HGDP00714	Cambodian	M51a1	M*(xM1,C,D)	E Eurasia/S Asia/Oceania
EU597566.1	HGDP00715	Cambodian	B5a1a	B4*(xB4a1,B2)/B5	E Eurasia
EU597577.1	HGDP00716	Cambodian	M17c	M*(xM1,C,D)	E Eurasia/S Asia/Oceania
EU597531.1	HGDP00788	Melanesian (Bougainville)	B4a1a1a1	B4a1	E Eurasia/Oceania
EU597543.1	HGDP00789	Melanesian (Bougainville)	Q1c	M*(xM1,C,D)	E Eurasia/S Asia/Oceania
EU597486.1	HGDP00792	Colombian	A2	A2*(xA2a,A2b)	Native America
EU597556.1	HGDP00807	Orcadian (Orkney)	X2b	X*(xX2a)	W Eurasia/Africa
EU597568.1	HGDP00808	Orcadian (Orkney)	H5b	H	W Eurasia
EU597579.1	HGDP00810	Orcadian (Orkney)	H5b	H	W Eurasia
EU597555.1	HGDP00823	Melanesian (Bougainville)	B4a1a1a	B4a1	E Eurasia/Oceania
EU597518.1	HGDP00945	Yakut (Siberia)	Z3a1	M*(xM1,C,D)	E Eurasia/S Asia/Oceania
EU597530.1	HGDP00946	Yakut (Siberia)	D5a2a2	D*(xD1,D3,D2a,D4h3a)	E Eurasia
EU597542.1	HGDP00947	Yakut (Siberia)	C5a1	C*(xC1,C4c)	E Eurasia
EU597498.1	HGDP00998	Karitiana (Brazil)	D1	D1	Native America
EU597510.1	HGDP01000	Karitiana (Brazil)	D1e	D1	Native America
EU597526.1	HGDP01028	Bantu_Herero (S-Africa)	L3d3a	L3*(xM,N)	Africa
EU597514.1	HGDP01033	Bantu_Zulu (S-Africa)	L0d1a1	L0k/L0d1a/L0d3	Africa
EU597502.1	HGDP01034	Bantu_Tswana (S-Africa)	L0d1b1	L0*(xL0k,L0d1a,L0d3)/L1	Africa
EU597533.1	HGDP01037	Pima (Mexico)	C1c1	C1*(xC1a)	Native America
EU597545.1	HGDP01039	Pima (Mexico)	C1b	C1*(xC1a)	Native America
EU597557.1	HGDP01040	Pima (Mexico)	C1b	C1*(xC1a)	Native America
EU597508.1	HGDP01075	Sardinian (Italy)	U5b3a1a	U*(xU6,U8b)	W Eurasia/S Asia
EU597520.1	HGDP01076	Sardinian (Italy)	J2b1a	J	W Eurasia
EU597532.1	HGDP01077	Sardinian (Italy)	H1	H	W Eurasia
EU597544.1	HGDP01147	North_Italian	U5a2b	U*(xU6,U8b)	W Eurasia/S Asia
EU597505.1	HGDP01224	Mongola (China)	B4a1c4	B4a1	E Eurasia/Oceania
EU597517.1	HGDP01225	Mongola (China)	C4a1	C*(xC1,C4c)	E Eurasia
EU597529.1	HGDP01226	Mongola (China)	A4a1a	A*(xA2)	E Eurasia
EU597538.1	HGDP01255	Mozabite (Algeria)	H	H	W Eurasia
EU597550.1	HGDP01256	Mozabite (Algeria)	V	HV0a [incl. V]	W Eurasia
EU597562.1	HGDP01257	Mozabite (Algeria)	U6a1a	U6	W Eurasia/Africa
EU597541.1	HGDP01327	She (China)	M7c1b	M*(xM1,C,D)	E Eurasia/S Asia/Oceania
EU597553.1	HGDP01328	She (China)	B4b1a2	B4*(xB4a1,B2)/B5	E Eurasia
EU597565.1	HGDP01329	She (China)	F4a1	R9 [incl. F]	E Eurasia
EU597576.1	HGDP01337	Naxi (China)	A4c1	A*(xA2)	E Eurasia
EU597494.1	HGDP01338	Naxi (China)	A11b	A*(xA2)	E Eurasia
EU597506.1	HGDP01339	Naxi (China)	B4a1	B4a1	E Eurasia/Oceania
EU597567.1	HGDP01377	French_Basque	HV0	HV*(xHV0a,H)	W Eurasia
EU597578.1	HGDP01378	French_Basque	T1a1	T	W Eurasia
EU597496.1	HGDP01379	French_Basque	K1a4a1	U8b [incl. K]	W Eurasia
EU597497.1	HGDP01402	Adygei (Russia_Caucasus)	U1a1	U*(xU6,U8b)	W Eurasia/S Asia
EU597509.1	HGDP01403	Adygei (Russia_Caucasus)	H1c	H	W Eurasia
EU597521.1	HGDP01404	Adygei (Russia_Caucasus)	H2a1	H	W Eurasia
EU597561.1	HGDP01408	Bantu (Kenya)	L2a1f	L2/L4/L6	Africa
EU597572.1	HGDP01411	Bantu (Kenya)	L1c2a1a	L0*(xL0k,L0d1a,L0d3)/L1	Africa
EU597490.1	HGDP01412	Bantu (Kenya)	L3b1a1	L3*(xM,N)	Africa

* According to Build 11 of the mtDNA tree available at http://www.phylotree.org [7].

## Conclusions

We developed an efficient and sensitive method for the multiplex genotyping of informative mtDNA SNPs, allowing for the inference of a person's matrilineal bio-geographic ancestry at a continental level. We would like to emphasize that matrilineal ancestry must be seen as reflecting only one aspect of the overall bio-geographic ancestry of a person [5,6,46]. A more accurate establishment of the overall bio-geographic ancestry is achievable when mtDNA is used in conjunction with informative Y-chromosomal (in the case of males) [8] and autosomal ancestry-informative DNA markers [47-50], especially when a person's biological ancestors are from different geographic regions resulting in mixed bio-geographic ancestry.

## Methods

### Reaction conditions

Multiplex PCR amplification was carried out in a reaction volume of 6 μL, containing 1x GeneAmp PCR Gold buffer (Applied Biosystems, CA, USA), 4.5 mM MgCl_2 _(Applied Biosystems), 100 μM of each dNTP (Roche, Mannheim, Germany), 0.35 units of AmpliTaq Gold DNA polymerase (Applied Biosystems), 0.001 to 1 ng genomic DNA template, and PCR primers (desalted; Metabion, Martinsried, Germany) in concentrations as specified in Tables 1, 2, 3. The reactions were performed in a Dual 384-well GeneAmp PCR System 9700 (Applied Biosystems) using optical 384-well reaction plates (Applied Biosystems), with the following cyclic conditions: 10 min at 95°C; followed by 30 cycles of 94°C for 15 s; 60°C for 45 s; and a final extension at 60°C for 5 min. PCR products were purified by adding 1.5 μL ExoSAP-IT (USB Corporation, OH, USA) to 6 μL PCR product, followed by incubation at 37°C for 15 min and 80°C for 15 min. Multiplex single-base primer extension was carried out in a reaction volume of 5 μL, containing 1 μL SNaPshot Ready Reaction Mix (Applied Biosystems), 1 μL purified PCR product and extension primers (HPLC-purified; Metabion, Martinsried, Germany) in concentrations as specified in Tables 1, 2, 3. The reactions were performed in a Dual 384-well GeneAmp PCR System 9700 (Applied Biosystems) using optical 384-well reaction plates (Applied Biosystems), with the following cycling conditions: 2 min at 96°C; followed by 25 cycles of 96°C for 10 s; 50°C for 5 s; and 60°C for 30 s. The reaction products were purified by adding 1 unit of Shrimp Alkaline Phosphatase (USB Corporation) to 5 μL of extension product, followed by incubation at 37°C for 45 min and 75°C for 15 min. PCR and extension primer details can be found in Table 1 for Multiplex 1, in Table 2 for Multiplex 2 and in Table 3 for Multiplex 3.

Extended primers were separated by capillary electrophoresis on a 3130xl Genetic Analyzer (Applied Biosystems) using POP-7 polymer by loading a mixture of 1 μL purified extension product, 8.8 μL Hi-Di formamide (Applied Biosystems) and 0.2 μL GeneScan-120 LIZ internal size standard (Applied Biosystems). Results were analysed using GeneMapper version 3.7 software (Applied Biosystems).

### Dilution series

For the purpose of sensitivity testing, genomic DNA from four individuals of different matrilineal continental origin was extracted from buccal swabs. For each individual, the DNA was diluted to obtain a solution of precisely 1 ng/μL as determined by two independent Quantifiler (Applied Biosystems) measurements. All Quantifiler assays were carried out according to manufacturer's recommendations. A dilution series was made from each of the four 1 ng/μL DNA solutions, producing concentrations of 0.25, 0.063, 0.016, 0.004 and 0.001 ng/μL for each individual. Concentrations of the dilutions were measured again and confirmed by triplicate Quantifiler measurements. The Quantifiler assays were carried out according to the manufacturer's recommendations, except for the addition of two extra dilutions to the recommended standard curve to be able to measure the very low DNA concentrations.

## Abbreviations

HVS: hypervariable segment; mtDNA: mitochondrial DNA; mtSNP: mitochondrial SNP; np: nucleotide position; NRY: non-recombining portion of the Y-chromosome; PCR: polymerase chain reaction; SNP: simple nucleotide polymorphism; STR: short tandem repeat.

## Competing interests

The authors declare that they have no competing interests.

## Authors' contributions

MVO designed the method and drafted the manuscript. MV tested and optimized the method. MK conceived the study and contributed to the manuscript. All authors read and approved the final manuscript.
